# Botswana country profile: Primary health care

**DOI:** 10.4102/phcfm.v18i1.5651

**Published:** 2026-09-12

**Authors:** Yaone Bogatsu, Ludo S. Monene, Mothusi Mogatle, Stephane Tshitenge, Billy Tsima, Kesegofetse Chabaesele, Keneilwe Motlhatlhedi

**Affiliations:** 1 Department of Family Medicine and Primary Health Care, School of Medicine, Faculty of Medicine and Health Sciences, University of Botswana, Gaborone, Botswana; 2 Department of Primary Care, Ministry of Health, Gaborone, Botswana; 3 Department of Primary Care, Ministry of Local Government and Traditional Affairs, Gaborone, Botswana; 4 School of Medicine, Faculty of Medicine and Health Sciences, University of Botswana, Gaborone, Botswana; 5 Global Health Office, Botswana Harvard Health Partnership, Gaborone, Botswana

**Keywords:** primary care, country profile, family medicine, Vision 2036, Universal Health Coverage, primary health care, community engagement, health systems

## Abstract

Botswana, an upper-middle-income country in sub-Saharan Africa (SSA), is sparsely populated, with a population of about 2.4 million. The country has a national roadmap to develop a world-class healthcare system. Achievements in health service delivery include access to healthcare, the national human immunodeficiency virus (HIV) programme and a foundation for primary health care (PHC). The government of Botswana spent approximately 14.6% of its general government expenditure on healthcare in 2022. Botswana’s Universal Health Coverage (UHC) Service index is lower than that of most middle-income countries and the global average. Family medicine (FM) is a relatively new speciality in Botswana; approximately 40 family physicians are practising in the public health system. The health workforce density in Botswana is 4.1 doctors and 31 nurses per 10 000 population. Primary health care in Botswana emphasises community engagement through district platforms. The strong PHC foundation is an opportunity for FM to contribute to high-quality health systems in Botswana.

## Introduction

Botswana is a landlocked country in sub-Saharan Africa (SSA) with an estimated population of 2.4 million people.^1^ The country is an upper-middle-income country with a democratically elected government.^2^ About 66% of the population resides in the cities, towns and urban villages.^1^ The country is sparsely populated, with a population density of 4.1 people per square kilometre. Botswana has a young population with a slight female preponderance. Table 1 summarises key demographic and socio-economic indicators according to the country’s 2022 population and housing census.^1^ Figure 1 shows the map of Botswana highlighting the distribution of public hospitals.^3^

**FIGURE 1:**
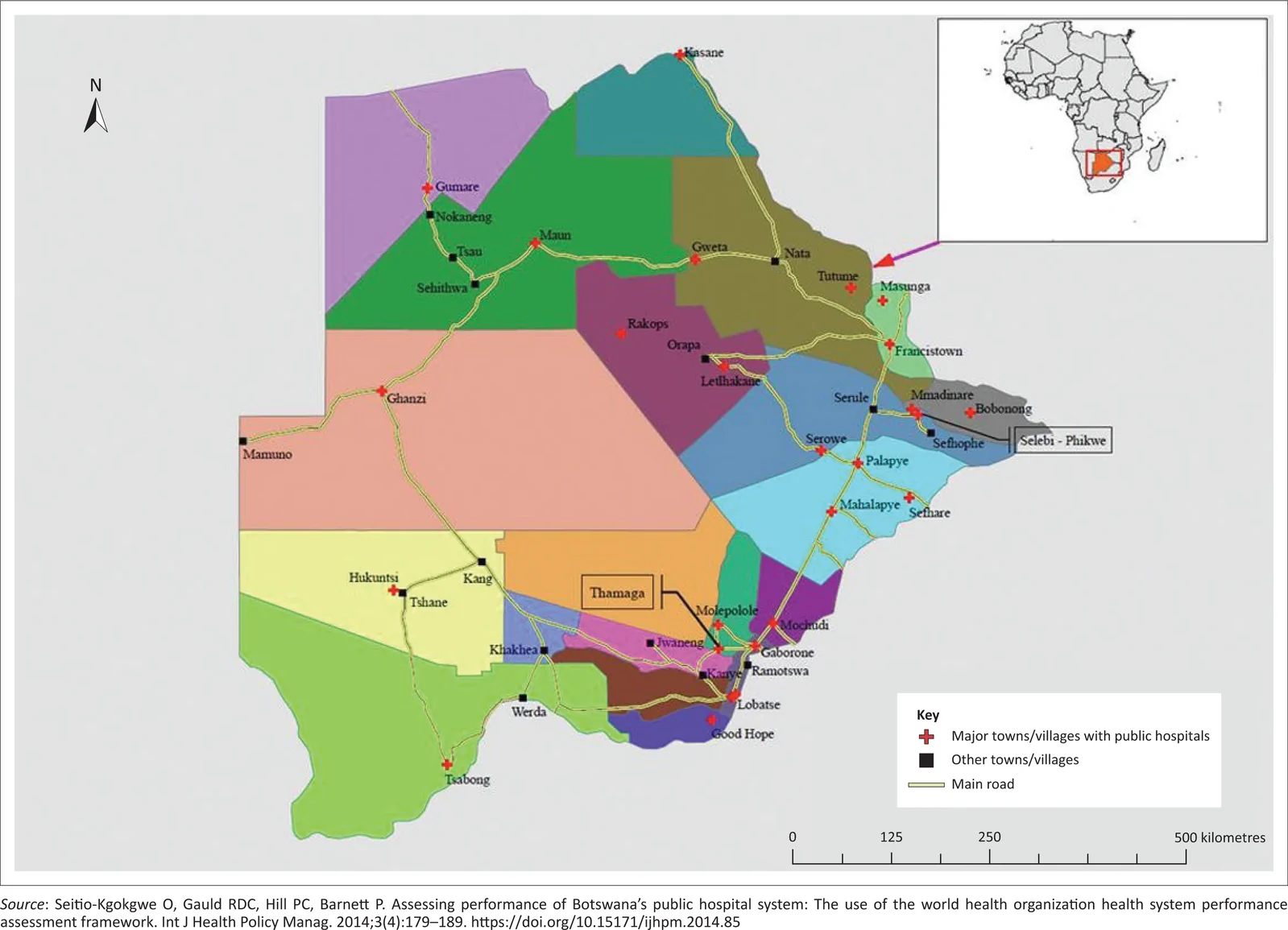
Map of Botswana showing villages with public hospitals, 2014.

**TABLE 1:** Demographic and socio-economic characteristics of Botswana, 2022.

Category	2022 Values
Population (2022 census)	~2.4 million
Median age	26 years
Rural: urban ratio	1:3
Crude birth rate	20.5 live births/1000
Crude death rate	6.7 deaths/1000
Infant mortality rate	27.9 deaths/1000
Life expectancy at birth	70.9 years
Literacy rates	88%
Unemployment rate	32.9%

*Source:* Adapted from Statistics Botswana. Population and Housing Census 2022. Statistics Botswana [homepage on the Internet]. Gaborone: Statistics Botswana; 2022 [cited 2025 Oct 19]. Available from: https://www.statsbots.org.bw; Statistics Botswana. Botswana Vital Statistics Report 2023 [homepage on the Internet]. Gaborone: Statistics Botswana; 2025 [cited 2025 Oct 20]. Available from: https://www.statsbots.org.bw

## Health service and disease profile

The national roadmap ‘Vision 2036’ aims towards ‘Achieving prosperity for all’ and aspires to a world-class healthcare service where Botswana will live long and healthy lives.^4^ Vision 2036 emphasises social inclusion, equity and access for all.^4^ However, there is a triple burden of disease with high human immunodeficiency virus (HIV) prevalence and increasing prevalence of non-communicable diseases and injuries, which may stall progress towards Vision 2036 and the achievement of Sustainable Development Goal 3 (SDG 3).^5^

## Health system inputs and structure

Notable achievements in health service delivery and outcomes for the country include better access to healthcare, the national HIV programme and a strong commitment to primary health care (PHC). The establishment of the country’s first medical school in 2009 aimed to strengthen human resources for health by augmenting the number of local doctors. However, the Ministry of Health (MoH) database recorded the density of doctors to be 4.3 per 10 000 population in 2012.^6^ The doctor density fluctuated between 3.4 and 4.6 from 2017 to 2023, with the highest being 4.6 in 2021, highlighting the need to heighten efforts to achieve the World Health Organization (WHO) doctor density threshold.^7^ In 2025, PHC was decentralised from the MoH to the Ministry of Local Government and Traditional Affairs, where it had previously been located until 2010. While this return of PHC to local government may strengthen community orientation and multisectoral engagement at the community and local government levels, the outcomes of this move are yet to be assessed.

## Healthcare expenditure and access

Botswana’s health expenditure is primarily government-based. The government of Botswana spent approximately 14.6% of its general government expenditure on healthcare in 2022, while out-of-pocket spending was less than 6% of total health expenditure.^2^ External funding contributed to less than 6% of health expenditure. However, it must be noted that an estimated 75% of this expenditure is allocated to secondary and tertiary care, while the remainder is divided between policymakers and primary care.^2^

The government of Botswana, through the MoH and the Ministry of Local Government and Traditional Affairs (LGTA), is the main provider of healthcare services in the country. This is done through a four-tiered health system: a network of nearly 1000 mobile health stops and 350 health posts linked to 311 clinics, 18 primary hospitals, two general referral hospitals, one quaternary hospital and one psychiatry referral hospital.^6^ The hospitals are under the MoH, while the Ministry of LGTA administers PHC, integrating district administration and primary care throughout the 31 health districts.^6^ Botswana’s Universal Health Coverage (UHC) Service index is lower than that of most middle-income countries and the global average. The low UHC service index is attributed to intermittent medication shortages and service fragmentation.

## Family medicine in Botswana

Family medicine (FM), a relatively new speciality in Botswana, is slowly gaining momentum as the clinical leaders of primary care. Currently, approximately 40 family physicians are practising in the public health system, covering over half of the districts. Since 2009, when the University of Botswana medical school was established, FM has been part of the medical school’s undergraduate curriculum with rotations in the 3rd and 5th years. Postgraduate FM training started in 2011 with 8 trainees,^8^ and by 2025, the programme had graduated 42 family physicians, adding to the few who were already working in the country. Family medicine training currently takes place at two rural sites: Mahalapye and Maun. The FM graduates are spread throughout the country, some functioning as family physicians in primary and district hospitals or clinics. Currently, 15 of the 18 primary hospitals, six of the nine district hospitals and both referral hospitals have a full-time FP. The scope of practice of family physicians in Botswana has been described elsewhere.^8^

## Service delivery

Primary care is mostly provided by nurses. The health workforce density in Botswana has declined, with 1003 doctors and 7641 nurses.^9^ In most districts, medical officers placed in primary care are based at either a central clinic, primary hospital or a district hospital, and conduct scheduled outreach visits to the clinics within the cluster. In districts with family physicians, the family physician may serve as a supervisor between these primary care providers and specialists or allied health providers.

Through this model, primary care facilities provide a wide range of health services, including preventive, promotive, curative and rehabilitative care across all ages. This is facilitated by staffing that includes the full range of primary care providers, including public health specialists and primary care providers.

The multisectoral approach of PHC is implemented through the representation of health in local government committees, as illustrated in Table 2. The table illustrates how the PHC team serves as a technical advisor by participating in various district committees. At the community level, there is the Village Development Committee (VDC), which oversees social development, including those related to health. Resolutions or recommendations from their forum are then channelled through to the community development and social services and/or health and education committees. Issues are then refined and escalated to the relevant offices within the district council or government.

**TABLE 2:** Ministry of Local Government and Traditional Affairs district council committees with a health portfolio.

District council committees with health portfolio	Leader	Role
**District level**		
District Development	District Commissioner	Monitors the implementation of development programmes/projects at community level
District Disaster Management	District Commissioner	Primary health care department participates as the technical advisor Coordinates disaster mitigation and response
District Health Planning	District Council Economic Planner	Aligns the district health plan to national health priorities and local resources
Community Development and Social Services Committee	District Council Social Development Officer	Monitors and promotes social development and protection at district level
Health and Education	District Council	Monitors and promotes integration of health and education in the pursuit of social development
District Multisectoral AIDS committee (DMSAC)	District Commissioner	HIV focused committee
Technical Advisory Committee	District Multisectoral AIDS committee	Encompasses all programmes geared towards HIV response
**Community level**		
Village Development (VDC)	VDC Chairperson	Oversees social development at the community level
Village Health (VHC)	VHC Chairperson	A sub-committee of the VDC, oversees health interventions and outcomes at community level

Note: The table was created by the authors (Yaone Bogatsu, Mothusi Mogatle & Keneilwe Motlhatlhedi) from information collected from several engagements with the members of the District council and the former district health management teams during the transition of Primary Care from Ministry of Health to Ministry of Local Government and Traditional Affairs. Author 3 (Mothusi Mogatle) sits in majority of the cases, they provided the role clarifications.

HIV, human immunodeficiency virus; VHC, village health committee; AIDS, acquired immunodeficiency syndrome; VDC, village development committee; DMSAC, district multisectoral AIDS committee.

As previously highlighted, our healthcare system operates under two parallel healthcare delivery models, that is, government and privately funded institutions. As noted in Table 2, there is a forum called ‘District Health Planning’, which serves as the convening point where both government-funded facilities and non-government/privately funded organisations meet to present individualised progress reports on their activities. This ensures alignment of various stakeholders with the national health priorities as directed by the District Economic Planning Team. Another forum where such interaction is illustrated is through the Disaster Management Forum, chaired by the District Commissioner, the most senior government representative in the district. This forum facilitates pulling of resources especially during natural hazards and major disease outbreaks.

Primary health care in Botswana has always emphasised community engagement through these district platforms. Different community-based services, that is, the home-based care programme, which is for patients on palliative care treatment, exist within our healthcare ecosystem. A clear pathway for interaction with local clinics has been established through a layered approach, starting with healthcare volunteers who visit individual patients and community healthcare educators who establish and participate in local health committees, that is, village health committee (VHC). These are committees that ensure a direct link between the community and the local clinic.

## Conclusion

Botswana is currently undergoing a health transition characterised by both epidemiologic changes and a shift in health service delivery models. While great strides have previously been made in health outcomes, the transition presents a challenge to the country’s vision of a long and healthy life for all. The strong PHC foundation is an opportunity for FM to contribute to high-quality health systems in Botswana.

## References

[1] Statistics Botswana. Population and housing census 2022 population of cities, towns and villages [homepage on the Internet]. 2022 [cited 2025 Oct 25]. Available from: https://www.statsbots.org.bw/sites/default/files/publications/Population%20%20Housing%20Census%202022-%20Key%20Demographic%20and%20Socio-Economic%20Indicators.pdf

[2] The World Bank Group. Data for Botswana, Upper middle income [homepage on the Internet]. 2025 [cited 2026 Feb 12]. Available from: https://data.worldbank.org/?locations=BW-XT

[3] Seitio-Kgokgwe O, Gauld RDC, Hill PC, Barnett P. Assessing performance of Botswana’s public hospital system: The use of the world health organization health system performance assessment framework. Int J Health Policy Manag. 2014;3(4):179–189. 10.15171/ijhpm.2014.85 PMC4181967 25279380

[4] Republic of Botswana. Botswana Vision 2036 overview: Achieving prosperity for all. Gaborone: National Planning Commission; 2022.

[5] United Nations. Transforming our world: The 2030 agenda for sustainable development [homepage on the Internet]. United Nations; 2015 [cited 2025 Oct 19]. Available from: http://sdgs.un.org/publications/transforming-our-world-2030-agenda-sustainable-development-17981

[6] Nkomazana O, Peersman W, Willcox M, Mash R, Phaladze N. Human resources for health in Botswana: The results of in-country database and reports analysis. Afr J Prim Health Care Fam Med. 2014;6(1):8. 10.4102/phcfm.v6i1.716 PMC4564932 26245420

[7] Statistics Botswana. Public health personnel statistical brief 2020 [homepage on the Internet]. 2020 [cited 2025 Oct 19]. Available from: https://www.statsbots.org.bw/sites/default/files/Health%20Personnel%20Statistical%20Brief%202020.pdf

[8] Tsima BM, Bogatsu Y, Motlhatlhedi K, Brockbank C, Ray SC, Tsima B. Family physicians as advocates for primary health care in Botswana. Afr J Prim Health Care Fam Med. 2025;17(1):a4908. 10.4102/phcfm.v17i1.4908 PMC12067618 40336414

[9] Statistics Botswana. Public health personnel 2023 [homepage on the Internet]. Gaborone: Statistics Botswana; 2025 [cited 2025 Oct 19]. Available from: https://www.statsbots.org.bw/sites/default/files/publications/PUBLIC%20HEALTH%20PERSONNEL%202025.pdf

